# Habitat change alters the expression and efficiency of a female ornament

**DOI:** 10.1093/beheco/arac080

**Published:** 2022-08-26

**Authors:** Sini Bäckroos, Lea Ala-Ilomäki, Ulrika Candolin

**Affiliations:** Organismal and Evolutionary Biology Research Programme, University of Helsinki, Helsinki, Finland; Organismal and Evolutionary Biology Research Programme, University of Helsinki, Helsinki, Finland; Organismal and Evolutionary Biology Research Programme, University of Helsinki, Helsinki, Finland

**Keywords:** environmental change, eutrophication, fecundity, reproduction, sexual selection, signals

## Abstract

Anthropogenic habitat changes are disrupting the mate choice process in a range of organisms, with consequences for populations and communities. Research has so far focused on male sexually selected traits and female mate choice, given their conspicuousness, whereas effects on female ornaments and male mate choice have been largely overlooked. Yet, females of many species develop ornaments that males use in mate choice. These ornaments can be costly and reduce female fecundity and viability and, hence, influence population growth rate. Thus, attention should be paid to changes in female ornaments and the consequences the changes have for populations. Here, we show that declining visibility in aquatic ecosystems reduces the investment of female three-spined stickleback in a melanin-based ornament in favor of increased mate search activity. The adjustment appears adaptive as males pay less attention to the ornament under poor visibility, and as melanin-based ornaments are generally physiologically costly. It is likely that past fluctuations in visibility have promoted the evolution of environment-dependent plasticity in female ornamentation. More attention should be paid to changes in female ornaments and their adaptive value, across taxa, given the impact that female investment in ornaments can have on fecundity and population growth rate. Environments are changing at an accelerating rate because of human activities and knowledge of the responses of both males and females is needed to evaluate and predict the ultimate impact on populations and biodiversity.

## Teaser

Females often use costly ornaments to attract males. Yet, how these are influenced by human-induced environmental change is unknown, although information on changes in both male and female traits are needed to determine the impact of environmental change on populations. We show that declining visibility reduces the efficiency of a female ornament in the threespine stickleback, and interestingly, that females show adaptive adjustments of the ornament to its reduced efficiency.

## INTRODUCTION

Habitats are changing across the world because of human activities. In many aquatic ecosystems, visibility is deteriorating because of anthropogenic eutrophication and enhanced growth of algae (Smith et al. 2006). Blooms of microalgae are reducing water clarity, whereas excessive growth of macroalgae – particularly of annual filamentous algae – is increasing the structural complexity of the habitats. This declining visibility influences the mating success of organisms that depend on vision for finding and evaluating potential mates (e.g. Järvenpää and Lindström 2004; Candolin 2009; Sundin et al. 2010; van der Sluijs et al. 2011; Alexander et al. 2017).

The mechanism behind altered mating success under poor visibility is often found to be reduced efficiency of male sexually selected visual signals (Candolin 2019). Poor visibility hampers the ability of males to attract females using visual signals, such as ornaments and courtship behaviors, whereas females experience difficulties in detecting the males and evaluating their qualities based on visual signals. A mechanism that has attracted less attention, but can have a decisive influence on mating success, is altered efficiency of female ornaments and their use in male mate choice. Female ornaments are usually less conspicuous than male ornaments, because of less intense competition among females for mates, and because ornaments are generally costly and investment in them can reduce female fecundity (Kokko et al. 2006; Lebas 2006; Fitzpatrick and Servedio 2018). Yet, female ornaments and male mate choice occur in a range of species alongside male ornaments and female mate choice (Amundsen 2000; Clutton-Brock 2009; Rubenstein and Lovette 2009; Tobias et al. 2012; Dale et al. 2015). Female ornaments are especially common in species where males have to be choosy because the number of females they can mate with is restricted, for instance because of costly parental care or female-biased operational sex ratio (Kokko and Johnstone 2002; Kokko et al. 2003; Servedio 2007; Fitzpatrick and Servedio 2018). Considering that ornaments are generally costly and that female fecundity and reproductive success determine potential population growth rate (Rankin and Kokko 2007), altered efficiency of ornaments, and female investment in them could influence population dynamics. Thus, knowledge on the effects of habitat changes on the expression and efficiency of female ornaments and their use in male mate choice is required for a thorough understanding of the effects that human-induced environmental changes have on populations.

A species where both males and females develop ornaments during reproduction is the three-spined stickleback (*Gasterosteus aculeatus*). Males build a tunnel-shaped nest and attract females to spawn by combining nuptial coloration with courtship, and then singly care for the eggs. The number of females that can spawn into a nest is restricted by the size of the nest and the ability of the male to care for the eggs by fanning fresh water into the nest (Wootton 1976). Thus, male mate choice occurs alongside female mate choice, with males preferring larger, more fecund females (Candolin and Salesto 2009). Females signal their readiness to spawn by developing an ornament; a bar-like pattern on the lateral sides of their body, which consists of darker, melanin-based vertical stripes against a silvery body coloration (Rowland et al. 1991). Females express the ornament only when carrying eggs ready to be spawned, and the ornament allows males to separate gravid females from males and females approaching the nest with the intention to destroy or raid it (Foster 1988). In addition, the ornament could facilitate the evaluation of the size of the female’s belly and her fecundity, as females adopt a head-up posture that exposes the abdomen to the male when interested in spawning (Rowland 2000). However, whether the ornament correlates with fecundity is unknown. Females benefit from expressing the ornament in the attraction of males (Rowland et al. 1991), but the expression is likely to incur costs, as melanin-based colors are generally costly, energetically, and by increasing predation and infection risks, as found in a range of species (Roulin 2016). Whether the stickleback ornament is costly has not been investigated, but appears likely as females express the ornament only for a few days when carrying eggs ready to be spawned. If the ornament is costly, females may have to balance ornament expression against other costly activities, such as mate searching that involves similar costs (energy expenditure and predation and infection risks), and against future fecundity and survival, as females can spawn repeatedly during one breeding season (Saarinen and Candolin 2020).

The benefits and costs of the female ornament could be environment dependent, as the benefit of ornaments depends on visibility (Endler 1992), whereas the cost of melanin-based colors depends on the availability of resources, resource needs, and predation and infection risks (Roulin 2016). Whether female stickleback adjust their investment in the ornament depending on environmental conditions is unknown, as very little attention has been paid to the ornament. Stickleback males reduce their investment in their ornament, red nuptial coloration, when visibility deteriorates (Candolin et al. 2016), but across species the pattern varies; some species adjust their ornaments to visibility, such as the brown trout *Salmo trutta* (Eaton and Sloman 2011) and the western rainbowfish *Melanotaenia australis* (Kelley et al. 2012), whereas others do not, such as the pipefish *Nerophis ophidion* (Sundin et al. 2016). These differences in responses are probably related to temporal and spatial variation in visibility and the evolutionary history of the species, which has determined the evolution of reaction norms for plastic responses (Chevin and Hoffmann 2017; Candolin 2019; Koneru and Caro 2022). The responses may be adaptive or not, depending on how they influence the costs and benefit of ornamentation. In general, maladaptive responses are likely when the conditions are evolutionary novel, as is often the case in human-disturbed environments (Sih et al. 2011; Tuomainen and Candolin 2011; Delhey and Peters 2017).

In the Baltic Sea, the breeding habitat of the three-spined stickleback is changing because of anthropogenic eutrophication (Andersen et al. 2017). Excessive growth of both micro- and macro-algae is reducing visibility, which hampers the ability of females to detect nesting males and evaluate their visual characteristics (Engström-Öst and Candolin 2007; Heuschele et al. 2009, 2012; Candolin and Vlieger 2013; Candolin et al. 2014). Whether the reduced visibility also influences the expression, efficiency, and signal value of the female ornament is unknown. If efficiency in terms of attracting males is reduced, the benefits of the ornament decrease, which may increase its relative cost. Efficiency may decrease if turbid water reduces the contrast between the darker bars and the lighter body coloration, or if filamentous algae create a visually noisy background that hampers the detection and evaluation of the bars. A reduction in signal value in indicating female fecundity or interest in spawning could again result in males choosing less fecund females, or females of lower genetic quality (less well adapted to the environment), or cause males to waste effort on courting unresponsive females, or even non-breeding individuals that approach the nest with the intention to raid it. Thus, changes in the expression, efficiency, and signal value of the female ornament could have repercussions for both female and male fitness and, hence, for the population and its future trajectory. We investigated if reduced visibility influences 1) female *investment* in the ornament, 2) the *signal value* of the ornament in reflecting female fecundity and inclination to spawn, and 3) the *efficiency* of the ornament in attracting males and, hence, the *adaptive value* of adjusting ornament expression to visibility. We performed two experiments that assessed the impact of altered habitat conditions and visibility on 1) the expression of the female ornament and its value as an indicator of female fecundity and interest in spawning, and 2) the efficiency of the ornament in attracting males.

## METHODS

We caught adult three-spined sticklebacks from shores close to Tvärminne Zoological Station in the Northern Baltic Proper (60°N, 23°), Finland, at the start of the breeding season, using Plexiglas traps (Candolin and Voigt 2001). We housed the fish in an outdoor facility, in large flow-through holding tanks under natural temperature and light conditions. We fed the fish defrosted chironomid larvae once a day. All procedures described were approved by the National Animal Experimental Board (ESAVI/5421/04.10.07/2016), and conducted according to national guidelines.

Individuals that came into reproductive condition – indicated by blue eyes in males and the swelling of the abdomen in females – were moved to separate tanks; females to large holding tanks and males to individual 10-L tanks. The male tanks contained a dish (15 cm in diameter) filled with sand and filamentous green algae for nest building and an artificial plant for shelter (Candolin 1997). To stimulate nest building, we showed the males a randomly selected gravid female, enclosed within a perforated, transparent plastic cup, twice a day for 15 min, until the male had built a nest and was used in the experiments. We used only males that built a nest within two weeks.

### Experiment 1. Female ornament expression and correlations with mate search activity and fecundity

To investigate if reduced visibility because of the growth of algae influences the expression of the female ornament, we moved females to individual tanks (35 × 40 cm, water depth 20 cm) with either an open or a vegetated habitat structure. In the open habitat, the bottom of the tank was covered by sand, whereas the vegetated habitat contained four bunches of artificial filamentous algae in addition to the sand. The artificial algae consisted of a cement clump to which 15 cm long polypropylene strings had been attached (Candolin et al. 2007). The vegetation was evenly distributed over the bottom so that about 75% of the area was covered. Filamentous algae reduce both light availability and impose a visually noisy background (Binzer et al. 2006; Kuczynski et al. 2020; Vadeboncoeur et al. 2021).

We used recently spawned females, who had released their first egg clutch within the last 12 h, to ensure that the whole egg maturation and ornament development period was spent under the experimental conditions. Females release their ovulated eggs spontaneously if no nest is available (Wootton 1976). Recently spawned females are easily recognized by their conspicuously contracted abdomen. The ornament fades immediately after spawning and is not expressed until the female has ovulated the next batch of eggs and these are ready to be spawned. We fed the females twice a day with defrosted chironomid larvae. When a female was ready to spawn, as indicated by a swollen abdomen and a dilated urogenital papilla through which the gelatinous mass of eggs was visible, we photographed the left lateral side of the female to measure ornament contrast, see description below.

To determine if ornamentation correlates with female investment in mate searching and fecundity, we allowed the females to search for and spawn with a nesting male. One hour after the photographing, we moved the female into an experimental arena (120 × 120 cm, water depth 20 cm). She was first enclosed in a transparent, perforated cylinder (diameter 20 cm) for 15 min to allow her to acclimatize to the new conditions, and then released by gently removing the cylinder, which lacked a bottom. At the opposite corner of the tank (diagonally) was a male in breeding condition, enclosed within a transparent, perforated cylinder (diameter 20 cm). He had been placed into the tank the day before, together with the dish containing his nest. The female could not see the male from where she was released, as visibility was restricted by four bunches of artificial algae that covered about 50% of the area, and by two clay pots (diameter 10 cm) that imitated larger rocks.

We recorded the latency of the female to approach the nesting male, within 5 cm of the cylinder, as this has been found to reflect investment in mate searching; females who invest less take longer to find a nesting male (Heuschele et al. 2012). We allowed the female to spend one minute by the male – during which time the female ornament darkened – before photographing her. After photographing, we released the female back into the experimental arena and removed the cylinder enclosing the male to allow the female to spawn with the male. Four females did not spawn within 2 h, and we replaced the males with new males, which were accepted by the females. After spawning, we removed the female and photographed her to measure changes in ornamentation during spawning. We recorded her body length (mm) and weight (g) and calculated body condition using the Fulton’s condition index: K = 100 000 × Weight/Length^3^. Two hours after spawning, when the eggs in the nest had hardened, we recorded the amount of eggs spawned by gently removing the clutch of eggs from the nest and weighing it to the nearest 0.01 g (Candolin 2000). Females from the two habitats did not differ in body length (*F*_1,58_ = 2.68, *P* = 0.11), body mass without eggs (*F*_1,58_ = 1.86, *P* = 0.18), or mass of eggs (*F*_1,58_ = 0.11, *P* = 0.75). We tested 30 females from each habitat treatment, 60 females in total.

### Ornament measurement

We placed the female into a small glass box containing a black sponge that restricted her movements, and photographed her under standardized light conditions (Candolin 1999). On the front side of the glass box was a black and white label to check that the light conditions were similar among images. We measured the visibility of the female ornament from the digital images as the contrast between the darker bars and the lighter body coloration, using the image analyzing software ImageJ (https://imagej.nih.gov/ij). We converted the images to grayscale and selected a constant sized area on the darkest bar and on the area to the right of the selected bar, and recorded the difference in darkness between these two areas. In addition, one of us (SB) used the images to visually rank females based on the conspicuousness of the ornament. The two methods correlated strongly (Pearson correlation: *N* = 20, *r* = 0.94, *P* < 0.001) and we used the software-based measure in the statistical analyses.

### Experiment 2. Male mate choice

To investigate if reduced visibility influences the preference of males for ornamented females, we allowed males to choose between two dummy females differing in ornamentation, under three visual conditions: clear water, poor visibility (mimicking an algal bloom), and visual background noise (mimicking the presence of filamentous algae). Thus, the treatments separated between the effects of altered visibility and altered background.

To initiate a trial, we moved a male with his nest (on the nesting dish) to an experimental tank (70 × 40 cm, water high 20 cm) in the evening before experimentation. The experimental tank had been divided into a male and a female section (both 35 × 40 cm) using a clear Plexiglas sheet. The backside of the female section was uniformly grey-green to mimic the natural background. In the poor visibility treatment, the Plexiglas sheet was covered with a green plastic film that made the environment on the other side look green while allowing the male to see through the sheet, imitating an algal bloom. In the visual background noise treatment, we added two bunches of artificial algae to the backside of the female compartment (Candolin et al. 2007).

The following morning, we placed two dummy females into the female section, one ornamented and one plain. We used several ornamented and plain dummies that slightly differed in form to separate out the influence of other characteristics than the ornament on male preferences. The dummies were about 7 cm long and realistically painted. The ornamented dummy had darker bars painted on both lateral sides, imitating an ornamented female. The two dummies hang from a transparent rod to which they were attached with transparent threads. The dummies were 23 cm from each other and 12 cm from the Plexiglas divider, and in front of the artificial vegetation in the background noise treatment. They were kept in a head-up posture that indicates readiness to spawn; a 45 degrees angle from the bottom (Rowland et al. 2002). We presented the two dummy females to the male for 15 min. An observer behind a blind gently moved the females to give the impression of living females. We noted which dummy female the male first approached within 5 cm, the number of times he visited each dummy, and the total time he spent by each.

We exposed each male to the three treatments sequentially, during three days, altering the order of the treatments among males. Environmental conditions were changed in the evening before the next day’s trial. Visibility in the spawning habitat of the stickleback can quickly change through the inflow of algal blooms and unattached filamentous algae. Thus, the treatments mimicked changes occurring in the natural habitat. We altered the position of the two dummies between treatments and replicates, and randomized the use of the individual dummies. We tested 37 males. No males or females were reused within or among experiments.

### Analyses

In experiment 1, we analyzed the effect of habitat on the expression of the ornament using a linear model (LM) with ornament contrast as response variable and habitat as fixed effect. To analyze the effect of habitat and ornamentation on latency to approach the nesting male, we used a LM with latency as response variable, habitat as fixed effect, and ornament as covariate. In order to assess if the expression of the ornament changed from maturation to the courtship stage, and then from courtship to the spawning stage (i.e., testing change between two stages), we used linear mixed models (LMMs) with ornament contrast at each stage as response variable, female identity as random effect, and habitat and stage as fixed effects. To assess if the ornament correlated with female traits (fecundity, size, and condition), we used LMs with ornament as response variable and female trait as covariate.

In experiment 2, we assessed the influence of habitat on which dummy female the male first visited (plain or ornamented) using a generalized linear mixed model (GLMM) with binomial distribution and male identity as random effect and habitat as fixed effect. To analyze the effect of habitat on the number of visits to each dummy female, we used a GLMM with Poisson distribution and log link, with number of visits to each female as response variable, male identity as random effect, and habitat and ornamentation as fixed effects. To assess the effect of habitat on the time spent by each dummy female, we used a LMM with time by each female as response variable, male identity as random effect, and habitat and ornamentation as fixed effects. We tested the assumptions of the models before analyses. All analyses were performed using the software IBM SPSS Statistics 26. The models and full estimates are given in the Supplementary Materials.

## RESULTS

### Experiment 1. Female ornament expression and correlations with mate search activity and fecundity

**Figure 1:**
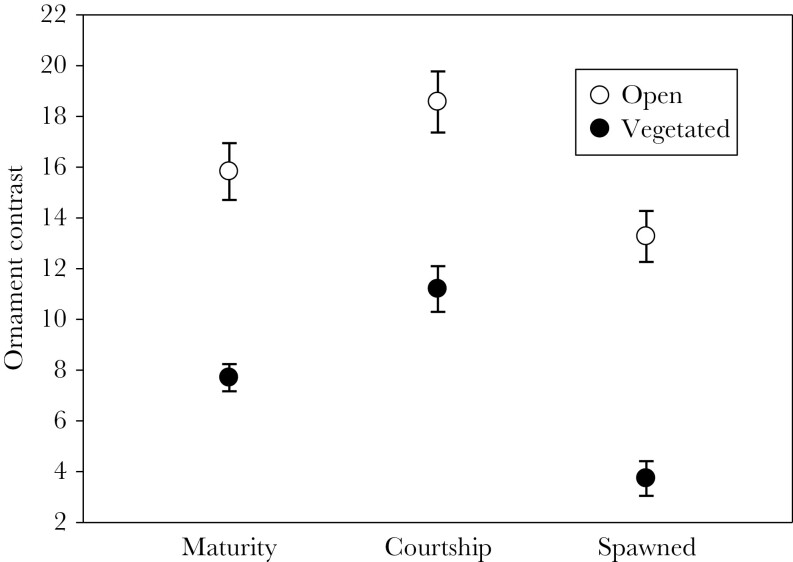
The ornamentation of females from open and vegetated habitats. Ornamentation was measured as the contrast between the darker bars and lighter body coloration, at three stages: 1) at maturity with eggs ready to be spawned, 2) after spending one minute with a courting male, and 3) after spawning. *N* = 30 for both female groups. Data show mean ± SE.

Females in the vegetated habitat developed less conspicuous ornaments than females in the open habitat (*N* = 30 females per habitat, *F*_1,58_ = 42.93, *P* < 0.001, Stage 1, “Maturity”, in Figure 1, Table S1).

**Figure 2:**
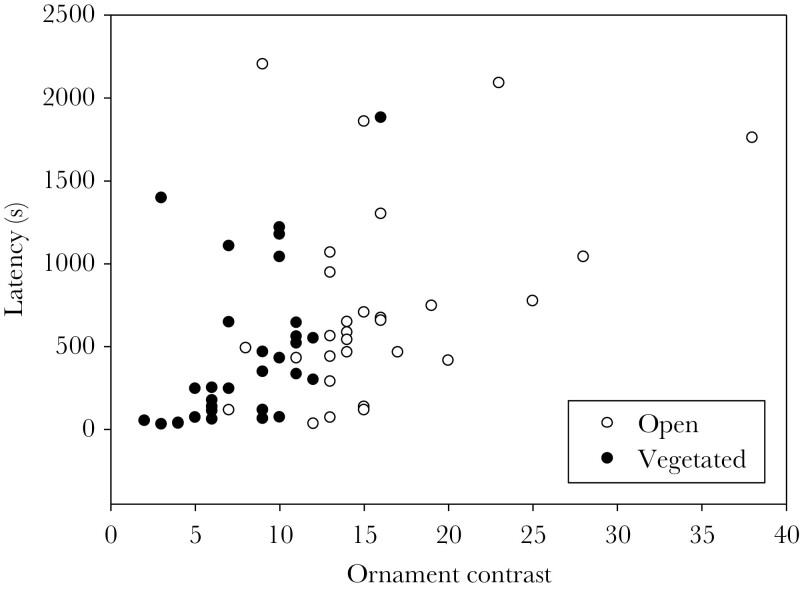
The relationship between ornamentation and latency to approach a nesting male, with females originating from an open or a vegetated habitat, *r* = 0.47, slope = 41.2 (SE = 10.1) when including all females. *N* = 30 per habitat treatment.

Females with more conspicuous ornaments took longer to approach the male, independent of the habitat they were moved from, which indicates that they invested less in searching for a nesting male (Table 1 and Table S2, Figure 2). The conspicuousness of the ornament increased after spending 1 min with the male (change in ornament contrast from maturity to courtship: *F*_1,58_ = 39.43, *P* < 0.001, Figure 1), independent of the past habitat of the female (interaction between habitat and change in ornamentation: *F*_1,58_ = 0.60, *P* = 0.44, Table S3). Females decreased ornamentation after spawning, with the decrease being more pronounced for females from the vegetated habitat (interaction between habitat and change in ornament from courtship to after spawning: *F*_1,58_ = 5.08, *P* = 0.028, Figure 1, Table S4).

**Table 1:** Influence of past habitat (open or vegetated) and female ornament contrast on latency to approach a nesting male. The non-significant interaction term was removed from the final model. A linear model was used to analyze the data. *N* = 30 per habitat treatment

	*F*_1,57_	*P*
Intercept	0.78	0.380
Habitat	0.13	0.724
Ornamentation	7.76	0.006
Habitat*ornamentation	0.01	0.937

The ornamentation of the females did not indicate their fecundity, neither absolute mass of eggs, nor relative mass of eggs (in relation to body mass without eggs), at any of the investigated stages (all *P* > 0.1, Table S5). Similarly, the ornament did not indicate body condition (all *P* > 0.1, Table S5).

### Experiment 2. Male mate choice

**Figure 3:**
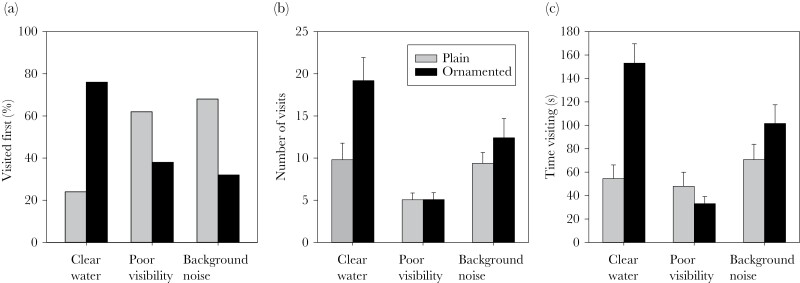
(a) Proportion of males visiting the plain or the ornamented dummy female first, (b) number of visits to the dummies (mean ± SE), and (c) time spent by them (mean ± SE), in the three treatments; clear water, poor visibility, and visual background noise. *N* = 37 males.

The interest of the male in the two dummy females depended on habitat treatment. Males visited the ornamented dummy female before the plain one in clear water, but not in the two other habitats (GLMM with binomial distribution, differences among habitats: *F*_2,108_ = 7.46, *P* = 0.001, *N* = 37 males, pairwise contrast between clear water and poor visibility: *t*_108_ = 3.54, *P* = 0.001, and between clear water and background noise: *t*_108_ = 4.13, *P* < 0.001, Figure 3a, Table S6). The number of visits to the two dummy females (Figure 3b) and the time spent by them (Figure 3c) also differed among the three habitats (interaction between habitat and ornament, Table 2, and Tables S7 and S8). Pairwise analyses show that males visited the ornamented female more often than the plain one in clear water (*F*_1,72_ = 108.10, *P* < 0.001) and in the background noise treatment (*F*_1,72_ = 15.46, *P* < 0.001), but not in the poor visibility treatment (*F*_1,72_ < 0.01, *P* = 0.96). Males spent more time by the ornamented dummy female in clear water (*F*_1,72_ = 23.61, *P* < 0.001), but not in the background noise (*F*_1,72_ = 2.25, *P* = 0.14) or the poor visibility (*F*_1,72_ = 1.22, *P* = 0.27) treatments.

**Table 2:** Influence of habitat and ornamentation of dummy females on number of visits to each dummy and time spent by them. To analyze number of visits, a GLMM with Poisson error term and log link was used. To analyze time spent by each dummy female, a LMM was used. In both models, male identity was added as random factor. *N* = 37 males

	Number of visits			Duration of visits		
	*F*	df	*P*	*F*	df	*P*
Intercept	92.71	5,216	<0.001	148.87	1,31	<0.001
Habitat	134.18	2,216	<0.001	13.55	2,185	<0.001
Ornament	45.94	1,216	<0.001	13.94	1,185	<0.001
Habitat*ornament	17.52	2,216	<0.001	10.34	2,185	<0.001

The total number of visits to the two dummy females (Figure 3b) and the total time spent by them (Figure 3c) also differed among the three habitats (Table 2). Pairwise contrasts show that total number of visits differed among all three habitats (all *P* < 0.001, see Table S7), with males visiting the dummy females most often in clear water and least often under poor visibility. Regarding total time spent by the dummy females, only the poor visibility treatment differed from the other two treatments (difference to poor visibility: *t*_216_ = 5.04, *P* < 0.001, and to background noise: *t*_216_ = 3.64, *P* < 0.001, Table S8), with males spending less time with the dummy females under poor visibility than in the two other treatments. There was no difference in time spent with the dummies between clear water and the background noise treatment (*t*_216_ = 1.40, *P* = 0.16, Table S8).

## DISCUSSION

The results show that females develop less conspicuous ornaments when visibility is poor and instead increase their investment in mate searching. The switch in investment appears adaptive as the ornament is less efficient in attracting males under poor visibility, and as increased search activity is needed to find nesting males when visibility is poor (Candolin and Voigt 1998; Heuschele et al. 2012).

The cause of the expression of weaker ornaments in the vegetated habitat with poor visibility could be higher costs than benefits of developing and expressing the ornament. Males paid less attention to the ornament when visibility was poor, which reduced its benefit. The perceived cost could again be higher as parasite abundance is greater in vegetated than in open habitats (Heuschele and Candolin 2010; Budria and Candolin 2015) and as melanin-based ornaments reduce immunocompetence (Roulin 2016).

The negative correlation between ornamentation and mate search activity, which occurred independent of the habitat the females had experienced during ornament development, suggests a trade-off in investment between the two traits; females that developed less visible ornaments invested instead in searching for males, and vice versa. An increased investment in search activity may be needed when the ornament is weak and less likely to attract males, while the costs of both traits may cause females to balance the traits against each other. Although we did not find the ornament to correlate with current female fecundity or body condition, it could influence future fecundity and body condition, as females in the present population can produce up to 7 egg clutches during one breeding season and as melanin-based colors generally incur both energetic and viability costs (Roulin 2016). The whole reproductive lifespan of the female needs to be included to assess possible costs of the ornament.

The efficiency of the ornament in attracting males depended on the habitat. The ornament attracted males in clear water, with males approaching the ornamented dummy female before the plain one, and visiting and spending more time with her, while poor visibility reduced ornament efficiency, as did visual background noise, although to a lesser degree. Males were also less attracted to the two dummies in the altered habitats, and especially under poor visibility. It is likely that males received less stimuli from the females when visibility was poor. Thus, reduced visibility influences male mate choice not only by hampering the ability of males to evaluate females, but also by reducing their interest in them. Similar results have been gained for female interest in ornamented males and the assessment of their qualities (Engström-Öst and Candolin 2007; Wong et al. 2007; Candolin et al. 2016). Thus, reduced visibility can influence mate choice by decreasing both male and female investment in mate choice. Mutual mate choice occurs in a range of species (Rosenthal 2017), and while effects of environmental change on female mate choice have repeatedly been demonstrated, little attention has been paid to effects on male mate choice (Candolin 2019).

We did not find the ornament to indicate female current fecundity or body condition, independent of habitat structure during egg maturation. The possibility still remains that the ornament indicates other qualities of females, such as the nutrient content of the eggs, or the genetic quality and viability of the expected offspring, as found for male ornaments (Candolin et al. 2016). Melanin-based ornaments correlate with various physiological traits, across taxa, particularly with immunocompetence and resistance to oxidative stress, and are often highly heritable (Cerenius et al. 2008; Ducrest et al. 2008; Roulin 2016; Cote et al. 2018). They are consequently often used as important indicators of individual qualities (Grether et al. 2004; D’Alba and Shawkey 2019). Alternatively, females could exploit sensory biases in males, if a more conspicuous ornament gives the impression of a bigger, more fecund female (Endler and Basolo 1998; Ryan and Cummings 2013). The ornament would then be a deceptive signal. More research is needed on the signal value of the ornament and why females differ in ornament expression.

The reduced efficiency of the ornament under poor visibility suggests that the recorded adjustment of the ornament to visibility is adaptive. It is likely that past selection has favored the evolution of plastic adjustment of the ornament to local conditions, as visibility naturally fluctuates in the breeding habitats of the investigated population, depending on spatial and temporal changes in algal blooms and the abundance of filamentous algae. Plasticity in relation to environmental conditions has been recorded also for sexually selected traits of male stickleback (Tuomainen et al. 2011; Candolin and Jensen 2021), and for males of several other species, although the adaptive values of these responses are often unknown (Candolin 2019; Fox et al. 2019). Our results suggest that past environmental conditions can have a decisive influence on the ability of both males and females to adjust their reproductive behavior in an adaptive manner to human-induced habitat changes (Sih et al. 2011; Tuomainen and Candolin 2011).

To conclude, an increasing number of studies show that females use ornaments to attract males, yet the sensitivity of the ornaments to habitat changes has remained unexplored (Candolin 2019). Considering that the investment of females in ornamentation can influence their fecundity and potential population growth rate (Fitzpatrick et al. 1995; Chenoweth et al. 2006), the topic would deserve more attention. We show that in the three-spined stickleback, females reduce their investment in a sexually selected ornament in favor of increased mate search activity when visibility is poor. The switch is most likely adaptive as poor visibility reduces the benefit of the ornament in mate attraction, and as melanin-based ornaments are generally costly and can reduce both viability and fecundity. Apparently past selection has promoted the evolution of adaptive plastic adjustments of ornamentation to visibility, given that visibility naturally fluctuates in the breeding habitat of the investigated population. Whether stickleback populations vary in their ability to adjust female ornaments to environmental changes depending on past conditions and evolutionary processes, as has been found for male ornaments (Boughman 2007; Tuomainen et al. 2011), is unknown, as is generally the case for female ornaments across taxa. More research should focus on the degree to which female ornaments and male mate choice are adjusted to changes in environmental conditions, and the consequences the responses have for reproductive success. Habitats are changing at an accelerating rate because of human activities, and the ability of species to adjust to these changes depends on responses of both males and females.

## Supplementary Material

arac080_suppl_Supplementary_Materials

## Notes

We thank Tvärminne Zoological Station for providing excellent working facilities.

Data availability: Analyses reported in this article can be reproduced using the data provided by Bäckroos et al. (2022).
