# Supplementary material for: Habitat change alters the expression and efficiency of a female ornament
Source: Behav Ecol. 2022 Aug 26;33(6):1133–40. doi: 10.1093/beheco/arac080 (PMC9735238; doi:10.1093/beheco/arac080)
Supplement: arac080_suppl_Supplementary_Materials [file arac080_suppl_supplementary_materials.pdf]

Supplementary Materials to

Habitat change alters the expression and efficiency of a female ornament

Sini Bäckroos, Lea Ala-Ilomäki, and Ulrika Candolin

Table S1 Impact of habitat on expression of the ornament at stage 1, when eggs ready to be spawned
LM: Ornament ~ Habitat

Source	Type III Sum of Squares	df	Mean Square	F	Sig.
Corrected Model	992,267[a]	1	992,267	42,934	,000
Intercept	8307,267	1	8307,267	359,443	,000
Habitat	992,267	1	992,267	42,934	,000
Error	1340,467	58	23,111		
Total	10640,000	60			
Corrected Total	2332,733	59			

R Squared = ,425 (Adjusted R Squared = ,415)

Table S2 Impacte of habitat and ornament contrast on latency to approach the nesting male
LM: Latency ~ Ornament + Habitat

Source	Type III Sum of Squares	df	Mean Square	F	Sig.
Corrected Model	3981955,764[a]	2	1990977,882	8,270	,001
Intercept	188472,722	1	188472,722	,783	,380
Ornament	1941754,164	1	1941754,164	8,065	,006
Habitat	30254,295	1	30254,295	,126	,724
Error	13723389,836	57	240761,225		
Total	39175940,000	60			
Corrected Total	17705345,600	59			

R Squared = ,225 (Adjusted R Squared = ,198)

Table S3. Impact of habitat and change from stage 1 to stage 2 on ornamentation

*LMM: Ornamentation ~ Habitat + Stage + Habitat*Stage + (1|female identity)*

Type III Tests of Fixed Effects[a]

Source	Numerator df	Denominator df	F	Sig.
Intercept	1	58	428,505	,000
Stage	1	58	39,427	,000
Habitat	1	58	36,238	,000
Stage * Habitat	1	58	,596	,443

Estimates of Covariance Parameters[a]

Parameter		Estimate	Std. Error
Residual		7,391092	1,372491
female	Variance	21,166092	4,667415

Table S4. Impact of habitat and change from stage 2 to stage 3 on ornamentation

*LMM: Ornamentation ~ Habitat + Stage + Habitat*Stage + (1|female identity)*

Type III Tests of Fixed Effects[a]

Source	Numerator df	Denominator df	F	Sig.
Intercept	1	58	547,888	,000
Stage	1	58,000	20,305	,000
Habitat	1	58	24,967	,000
Stage * Habitat	1	58,000	5,076	,028

Estimates of Covariance Parameters[a]

Parameter		Estimate	Std. Error
Residual		18,445402	3,425225
female	Variance	9,618391	3,895377

Table S5. The dependence of female ornamentation at three stages – maturity, after courtship, and after spawning - on fecundity (absolute mass of eggs), and relative fecundity (mass of eggs in relation to body mass without eggs), and body condition (calculated as Fulton's condition index K).

LM: 'Female trait'~ Ornamentation

Ornamentation	Fecundity				Relative fecundity				Condition			
	r^2	SE	$F_{1,58}$	P	r^2	SE	$F_{1,58}$	P	r^2	SE	$F_{1,58}$	P
Maturity	0.01	6.30	0.80	0.38	<0.01	6.33	0.15	0.70	<0.01	6.34	<0.01	0.94
After courtship	<0.01	6.92	0.24	0.62	0.01	6.89	0.63	0.43	<0.01	6.93	<0.01	0.93
After spawning	0.04	5.00	2.24	0.14	0.02	5.05	0.89	0.35	<0.01	5.08	0.21	0.65

Adding the habitat in which the female developed the eggs (open or vegetated) to the models did not influence the results and was deleted from the models

Table S6. The impact of habitat treatment on which dummy female the male first visited, ornamented or non ornamented

GLMM with binomial probability distribution: First visited ~ habitat+ (1|male identity)

Fixed Effects[a]

Source	F	df1	df2	Sig.
Corrected Model	7,469	2	108	,001
Habitat	7,469	2	108	,001

Probability distribution: Binomial, Link function: Logit

Pairwise Contrasts

Pairwise Contrasts	Contrast Estimate	Std. Error	t	df	Adj. Sig.	95% Confidence Interval Lower	95% Confidence Interval Upper
C - T	-,377	,107	-3,544	108	,001	-,589	-,166
C - V	-,431	,104	-4,127	108	7,248E-5	-,638	-,224
T - C	,377	,107	3,544	108	,001	,166	,589
T - V	-,054	,111	-,485	108	,629	-,273	,166
V - C	,431	,104	4,127	108	7,248E-5	,224	,638
V - T	,054	,111	,485	108	,629	-,166	,273

Treatment, T=turbid, V=vegetation, C=clear

Table S7. The impact of habitat treatment and dummy female ornamentation on number of visits to each dummy female

GLMM with Poisson probability distribution: Number of visits ~ habitat + ornamented + (1|male identity)

Fixed Effects[a]

Source	F	df1	df2	Sig.
Corrected Model	92,713	5	216	,000
Habitat	134,178	2	216	,000
Ornamented	45,941	1	216	,000
Habitat * Ornament	17,524	2	216	,000

Probability distribution: Poisson, Link function: Log

Random Effect

Random Effect Covariance	Estimate	Std. Error	Z	Sig.	95% Confidence Interval	
					Lower	Upper
Var(Male)	,344	,093	3,723	,000	,203	,583

Covariance Structure: Variance components

Pairwise Contrasts

Pairwise Contrasts	Contrast Estimate	Std. Error	t	df	Adj. Sig.	95% Confidence Interval	
						Lower	Upper
C - T	7,482	,890	8,410	216	5,551E-15	5,728	9,235
C - V	2,537	,570	4,452	216	1,363E-5	1,414	3,661
T - C	-7,482	,890	-8,410	216	5,551E-15	-9,235	-5,728
T - V	-4,944	,649	-7,624	216	7,692E-13	-6,223	-3,666
V - C	-2,537	,570	-4,452	216	1,363E-5	-3,661	-1,414
V - T	4,944	,649	7,624	216	7,692E-13	3,666	6,223

Treatment, T=turbid, V=vegetation, C=clear

Table S8. The impact of habitat treatment and dummy female ornamentation on number of visits to each dummy female

LMM: Time of visits ~ habitat + ornamented + (1|male identity)

Fixed Effects[a]

Source	F	df1	df2	Sig.
Corrected Model	12,345	5	216	,000
Habitat	13,552	2	216	,000
Ornament	13,935	1	216	,000
Habitat * Ornament	10,343	2	216	,000

Random Effect

Random Effect Covariance	Estimate	Std. Error	Z	Sig.	95% Confidence Interval	
					Lower	Upper
Var(Male)	447,894	339,920	1,318	,188	101,199	1982,334

Covariance Structure: Variance components

Pairwise Contrasts

Pairwise Contrasts	Contrast Estimate	Std. Error	t	df	Adj. Sig.	95% Confidence Interval	
						Lower	Upper
C - T	63,243	12,538	5,044	216	9,648E-7	38,530	87,956
C - V	17,622	12,538	1,405	216	,161	-7,091	42,334
T - C	-63,243	12,538	-5,044	216	9,648E-7	-87,956	-38,530
T - V	-45,622	12,538	-3,639	216	,000	-70,334	-20,909
V - C	-17,622	12,538	-1,405	216	,161	-42,334	7,091
V - T	45,622	12,538	3,639	216	,000	20,909	70,334

Treatment, T=turbid, V=vegetation, C=clear.
